# Supplementary material for: Predicting European cities’ climate mitigation performance using machine learning
Source: Nat Commun. 2022 Dec 5;13:7487. doi: 10.1038/s41467-022-35108-5 (PMC9723121; doi:10.1038/s41467-022-35108-5)
Supplement: Supplementary file 1 — Supplementary Information [file 41467_2022_35108_MOESM1_ESM.pdf]

Supplementary Information

Predicting European Cities' Climate Mitigation Performance using Machine Learning

Angel Hsu[1,2,3], Xuewei Wang[1,2,3], Jonas Tan[4], Wayne Toh[4], and Nihit Goyal[4\5]

[1]Department of Public Policy, University of North Carolina-Chapel Hill, Abernethy Hall, Chapel Hill, NC, 27599

[2]Data-Driven EnviroLab, University of North Carolina-Chapel Hill, Chapel Hill, NC 27599

[3]Institute for the Environment, University of North Carolina at Chapel Hill, United States of America

[4]Yale-NUS College, Singapore, 10 College Ave W, 138609

[5]Faculty of Technology, Policy and Management, TU Delft, Building 31 Jaffalaan 5, 2628 BX Delft, Netherlands

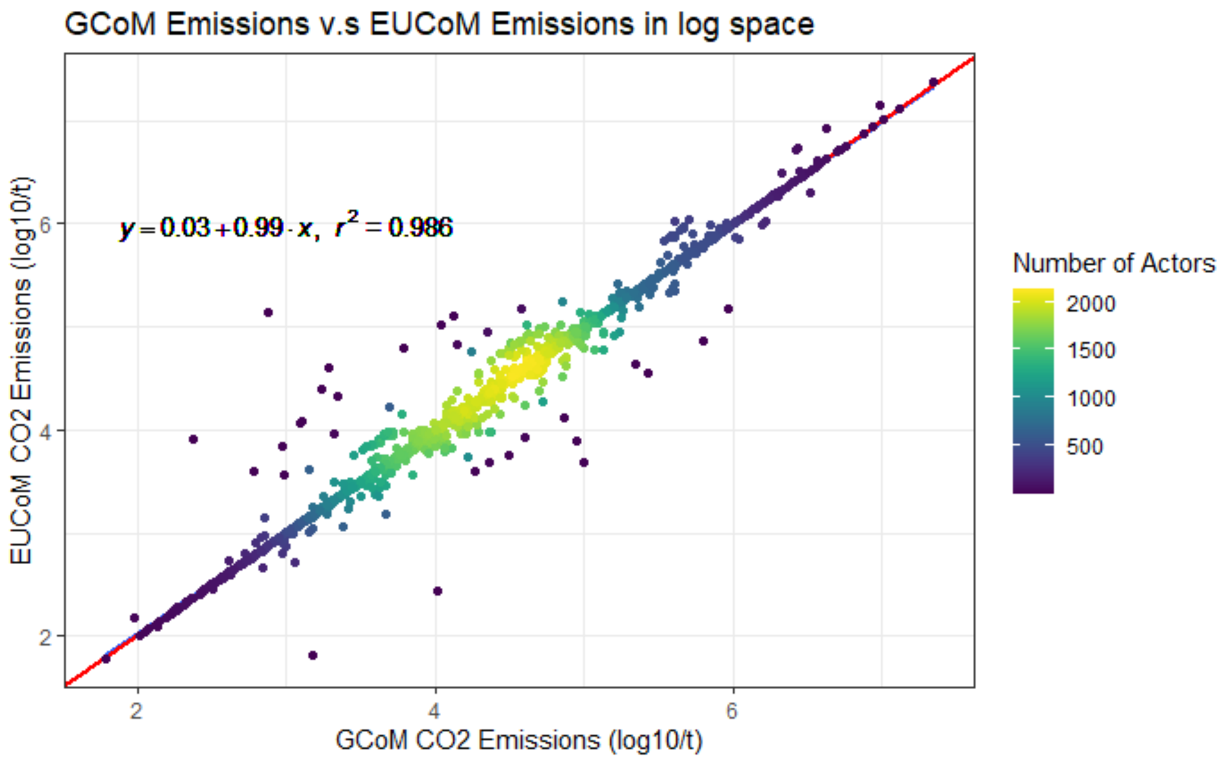

Figure S1. Comparison of logged emissions data points between the two primary self-reported emissions data from Kona et al. (2021) and the EU Covenant of Mayors for Climate and Energy website. Where a city had self-reported emissions data in both Kona et al. (2021) and the EUCoM website for the same years, we prioritized data from Kona et al. (2021), since they provide a series of validation and checks on these data.

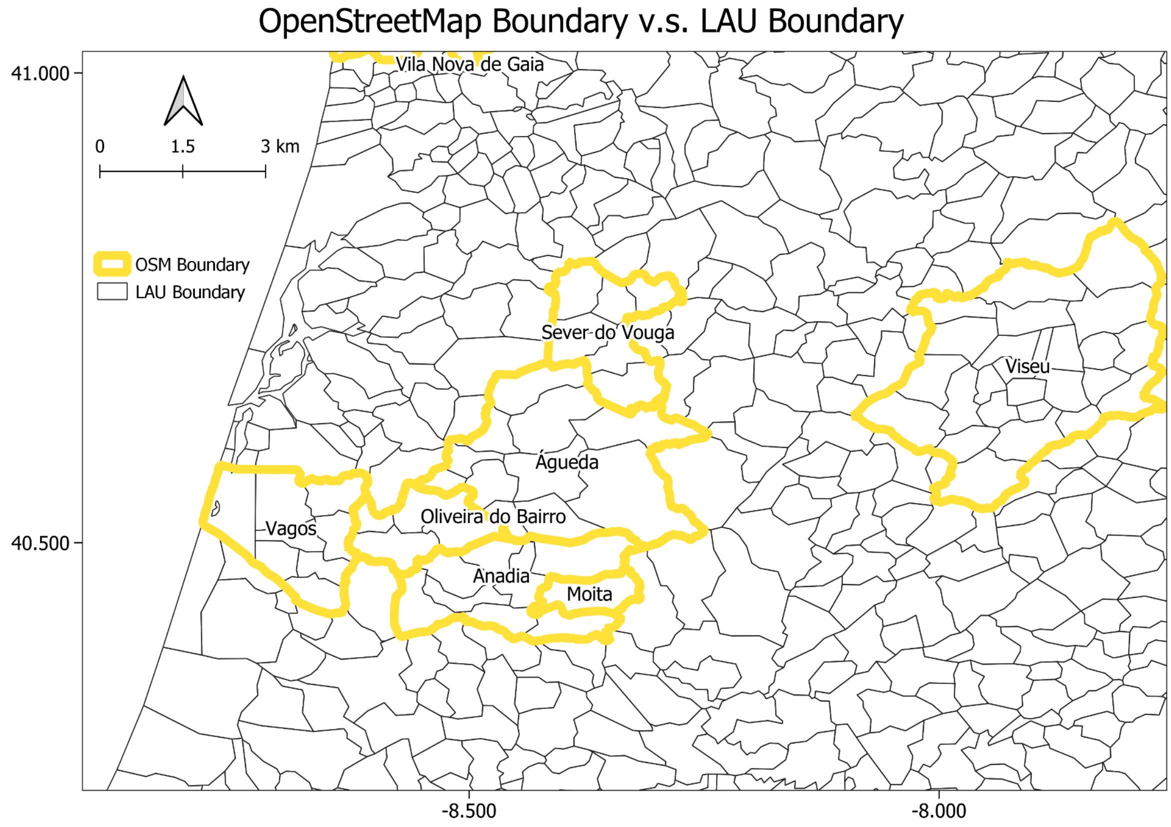

Figure S2. Comparison of city boundaries from OpenStreetMap (yellow polygons) vs. LAU Units (black polygons) in Portugal. (Base map source: OpenStreetMap, 2021).

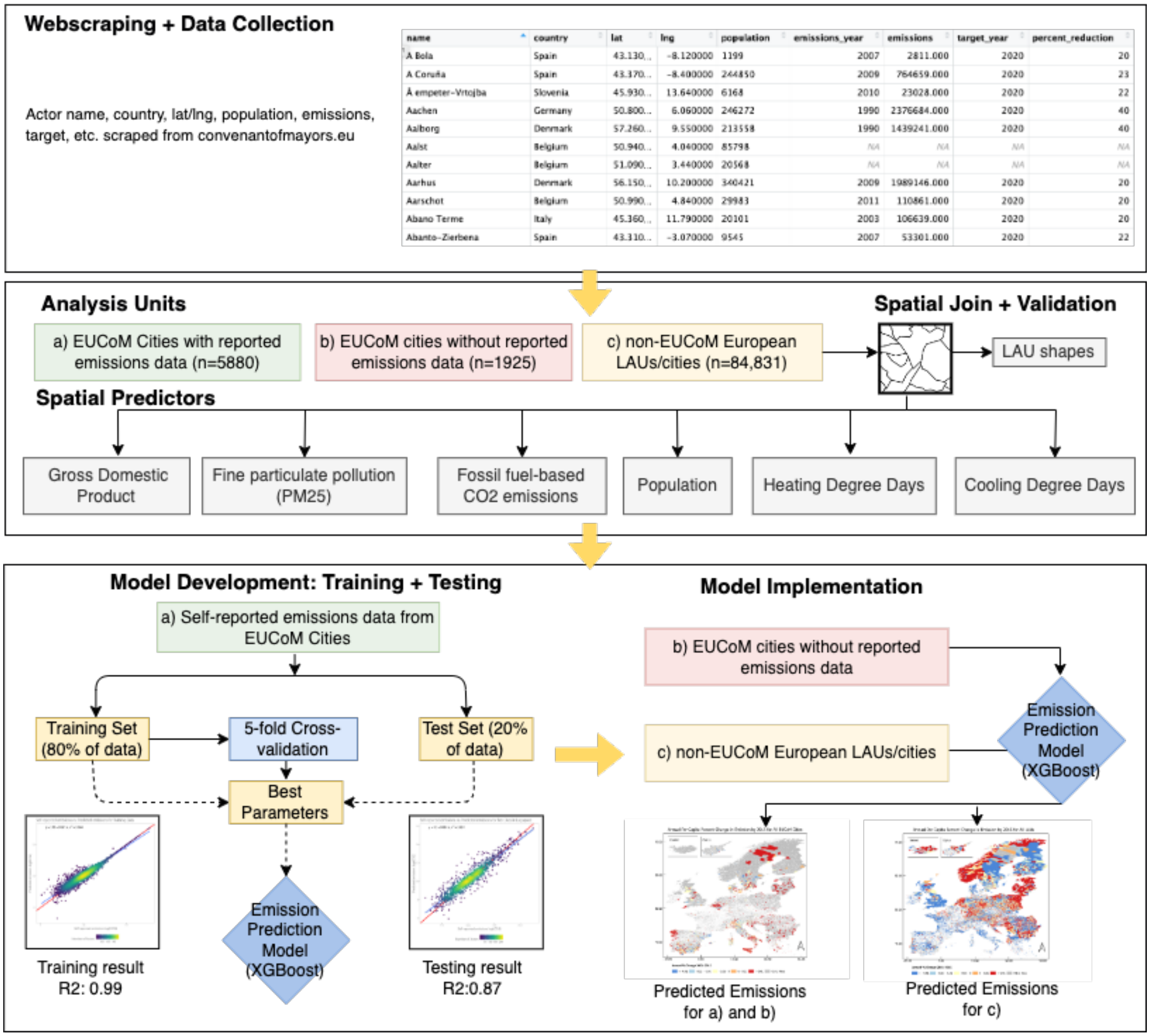

Figure S3. Overview of methodological workflow and data processing steps. Source: authors.

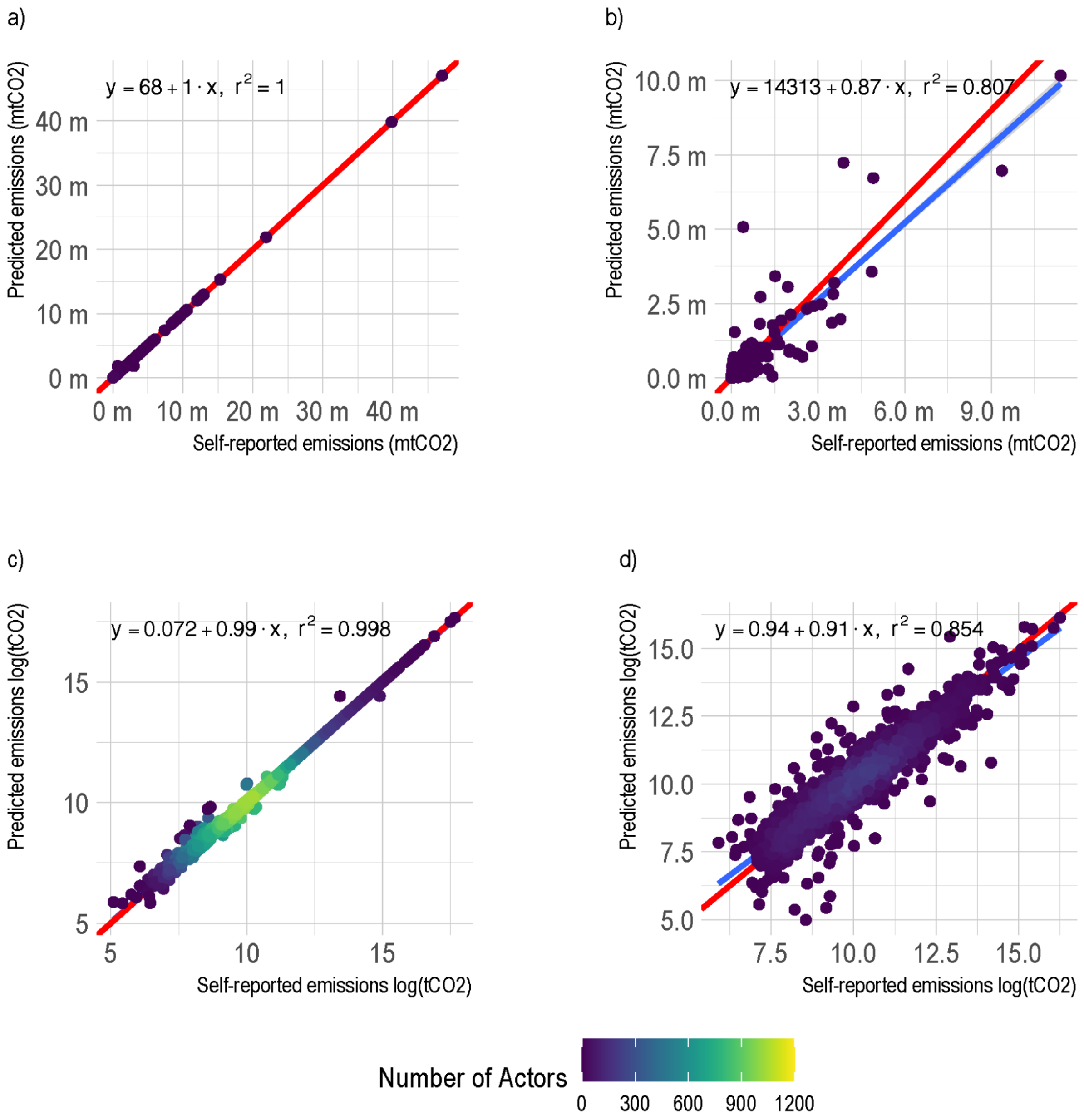

Figure S4. Scatterplots comparing self-reported emissions and emissions predicted by the model for both training (80 percent) and test (20 percent) datasets in both normal and log space. Panel a shows self-reported emissions compared to predicted emissions in normal space for the training dataset; Panel b shows self-reported emissions compared to predicted emissions in normal space for the test dataset; Panel c shows self-reported emissions compared to predicted emissions in log space for the training dataset; and Panel d shows self-reported emissions compared to predicted emissions in log space for the test dataset.

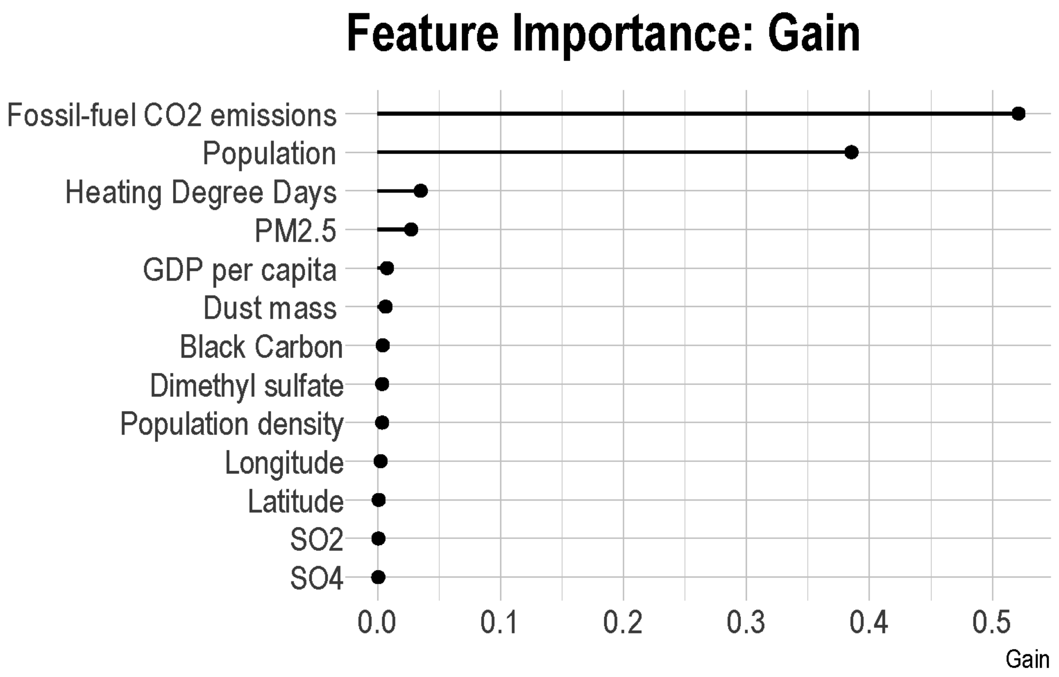

Figure S5. Importance of various predictor variables to the emissions model when all variables considered are included in the model.

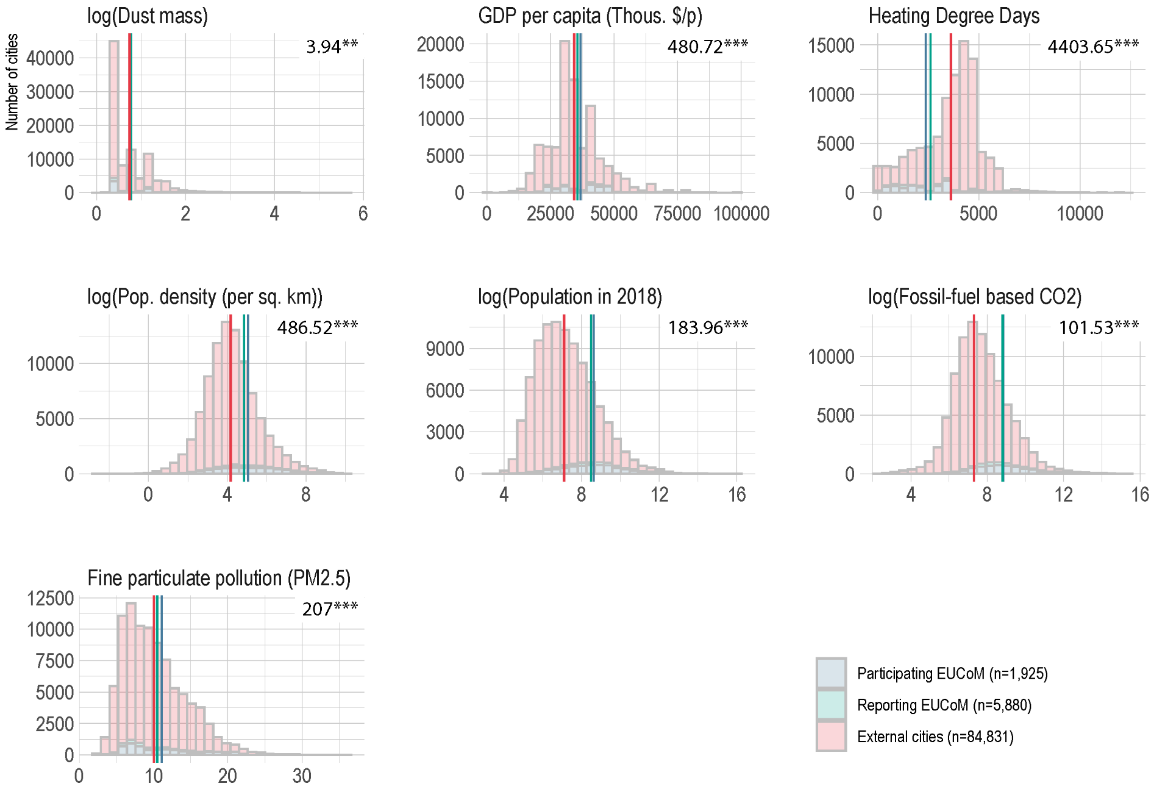

Figure S6. Histograms comparing attributes of the study's three groups of cities 1) reporting (n=5,580); participating (n=1,925) and external cities (n=84,831). Krustal-wallis test statistics and significance comparing differences in group means reported in the top right-hand corner of each panel. Note: *p<0.1; **p<0.05; ***p<0.01.

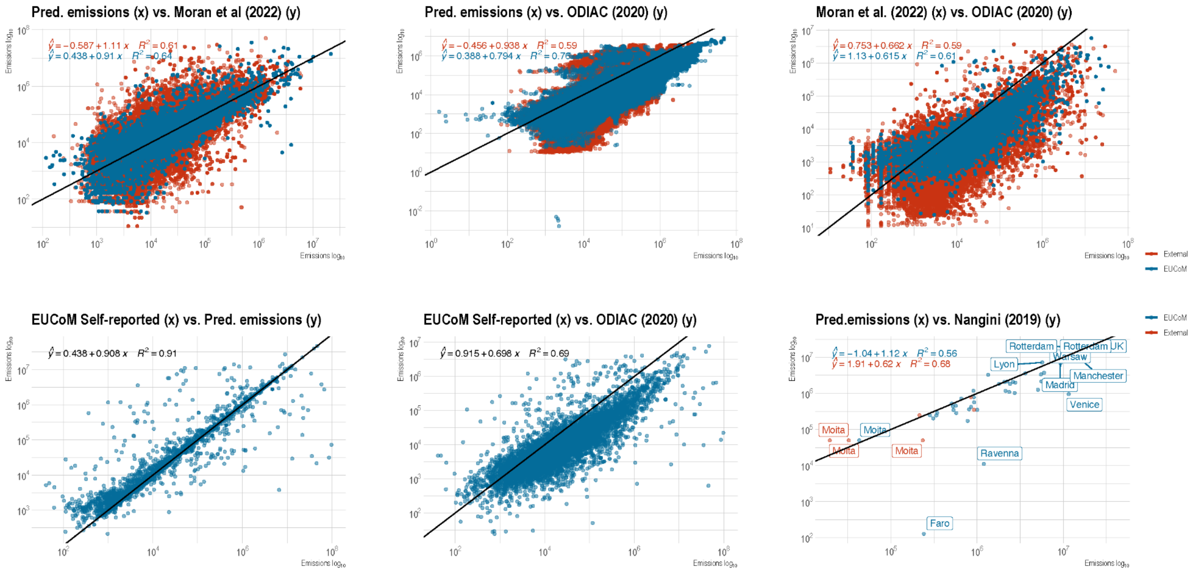

Figure S7. Comparisons and validation of the predicted emissions data in our study and other studies estimating city carbon emissions Moran et al. (2022) and Nangini et al. (2019), and other datasets (Oda, 2020; and Kona et al.'s (2021) harmonized EUCoM dataset.

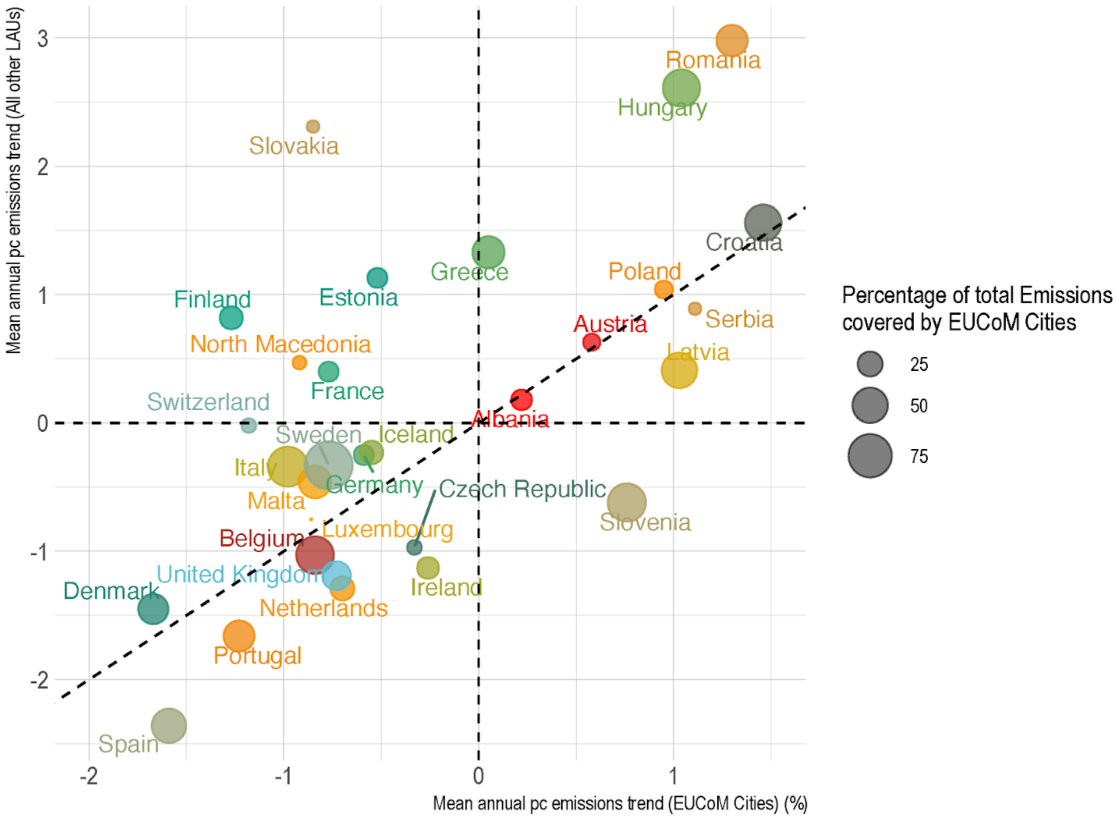

Figure S8. Mean annual per capita emissions reduction (%) of EUCoM cities versus mean annual per capita emissions trend of all other local administrative units (LAUs), sized by the percentage of total national emissions covered by cities participating in the EUCoM. Points are shaded by country.

Supplementary Table 1. Predictor variables included in machine-learning model

**Variable - long name**	**Variable - short name**	**Definition**	**Temporal Resolution**	**Spatial Resolution**	**Source**
Heating and Cooling Degree Days	HDD / CDD	Number of monthly-averaged temperature estimates that deviate from a baseline temperature	2000-2018	0.65x0.5 degree (72.15x55 km)	NASA MERRA-2 - satellite derived and processed[46]
Fossil-fuel-based CO2 emissions	ODIAC	Emissions arising from combustion of fossil-fuels, cement production and gas flaring	2000-2018	1 km	ODIAC (Oda, 2020)[45]
Fine-particulate air pollution	PM25	Exposure to fine particulate air pollution	2001-2018	~1 km	Van Donkelaar et al (2020)[47]
Population	POP	Annual interpolated population	2000-2020, in 5-year increments	1 km	CIESIN (2018)[50]
Population density	POP_DENSITY	Annual interpolated population density divided by area in square km	Original data is from 2000-2020, in 5-year increments.	30 arc-second (approximately 1 km at the equator)	CIESIN (2018)[50]
Dust mass concentration	DUST	Dust surface mass concentration	2000-2020, monthly measurements averaged to annual values	0.5 ° x 0.625 ° (55km*70km).	NASA MERRA-2 - satellite derived and processed[46]
Gross Domestic Product	GDP	Gross Domestic Product per capita	1990 to 2015	1 km	Kummu et al., (2018)[51]

Supplementary Table 2. Summary Statistics 1) Cities reporting emissions data in the European Covenant of Mayors for Climate and Energy (EUCoM); 2) Cities not reporting emissions data in the EUCoM; 3) All other European LAUs. Data correspond to year 2018.

Statistic	N	Min	Pctl(25)	Median	Pctl(75)	Max	Mean	St. Dev.
(1) EUCoM Cities reporting emissions data								
Dust	5,855	1.047	1.513	1.583	2.980	180.625	2.549	4.472
GDP per capita	5,565	6,153.805	28,711.190	39,553.600	44,356.970	82,240.430	36,814.960	9,487.952
Heating degree days	5,880	0.000	1,095.098	2,070.117	3,365.766	8,894.994	2,376.842	1,522.528
Population density	5,880	0.238	46.333	151.551	513.086	20,340.250	575.727	1,376.272
Population	5,880	35.308	1,704.513	5,279.799	15,319.120	8,965,276.000	32,719.970	181,348.900
Fossil-fuel CO2 emissions	5,874	0.000	2,358.373	5,978.346	17,143.030	5,705,648.000	40,550.020	201,245.200
Fossil-fuel CO2 emissions per capita	5,874	0.000	0.799	1.220	1.933	775.742	2.257	13.889
Fine particulate matter pollution (PM2.5)	5,841	2.151	7.040	9.912	14.360	29.181	11.114	4.889
(2) EUCoM Cities not reporting emissions data								
Dust	1,885	1.361	1.528	1.618	3.087	59.279	2.534	2.431
GDP per capita	1,769	7,476.400	27,972.810	35,271.890	42,658.540	89,288.440	35,558.130	9,237.782

Heating degree days	1,925	0.000	1,189.659	2,789.184	3,654.686	7,956.604	2,619.353	1,640.919
Population density	1,925	2.344	43.684	112.618	322.863	17,795.790	471.680	1,335.870
Population	1,925	22.149	1,460.741	4,013.025	12,602.690	2,672,758.000	35,317.960	171,628.400
Fossil-fuel CO2 emissions	1,924	0.000	2,321.341	5,327.714	17,481.530	4,199,135.000	42,027.260	198,415.700
Fossil-fuel CO2 emissions per capita	1,924	0.000	0.899	1.383	2.199	54.351	1.861	2.139
Fine particulate matter pollution (PM2.5)	1,895	2.924	7.369	9.401	13.290	30.334	10.494	4.050
(3) All other LAUS								
Dust	84,570	1.057	1.497	1.677	2.843	290.207	2.488	3.890
GDP per capita	83,493	1,319.210	28,983.290	32,744.380	39,935.240	99,833.490	34,293.130	10,475.820
Heating degree days	84,831	0.000	2,800.095	3,952.717	4,545.292	12,371.250	3,625.570	1,464.376
Population density	84,831	0.090	28.133	60.216	135.874	29,391.080	206.521	748.560
Population	84,831	29.914	407.897	1,013.053	2,946.106	866,314.800	4,433.324	16,869.540
Fossil-fuel CO2 emissions	84,716	0.000	748.764	1,681.687	4,306.638	3,600,398.000	7,473.031	54,160.930
Fossil-fuel CO2 emissions per capita	84,716	0.000	1.000	1.617	2.519	2,659.053	2.242	14.397
Fine particulate matter pollution (PM2.5)	84,170	1.915	6.794	9.306	12.462	35.632	10.052	4.162

Supplementary Table 3. Summary performance statistics of cities participating in the EUCoM.

country	Number of EUCoM cities evaluated	population	Share of national emissions (%)	Share of national population (%)	On track (%)	Reporting emissions (%)	Percentage of cities reducing emissions (%)
Austria	21	110923±388289	15	26	19	62	57
Belarus	10	153961±131148	9	16	0	30	10
Belgium	473	24077±68467	56	99	47	66	86
Bulgaria	26	100418±246950	40	37	23	88	31
Croatia	63	24992±63433	53	39	11	89	35
Czech Republic	19	121749±307041	9	22	37	32	47
Denmark	49	75784±95084	46	64	80	92	94
Estonia	5	100626±159730	16	38	20	100	60
Finland	19	143122±139430	23	49	53	68	89
France	114	117653±270325	18	20	51	71	68
Germany	81	222695±447453	16	22	35	54	60
Greece	156	38113±107225	42	55	24	83	49
Hungary	162	58975±274522	55	98	19	38	27
Ireland	16	111873±164629	15	36	50	69	50

Italy	3,930	14110±84543	64	92	51	77	68
Latvia	20	66237±157486	50	69	30	95	40
Lithuania	15	71618±102545	28	39	13	80	27
Luxembourg	9	2943±1427	2	4	44	11	67
Malta	23	5907±4601	42	27	48	83	70
Netherlands	31	164848±179757	23	29	32	55	74
Norway	10	216826±238756	31	41	70	40	80
Poland	46	172670±375171	13	21	11	80	46
Portugal	140	24779±58851	38	34	56	78	75
Romania	100	157493±434528	39	81	10	65	26
Slovakia	29	20169±49424	7	11	59	10	66
Slovenia	31	44901±89418	58	67	10	97	45
Spain	2,114	19780±108487	50	89	68	80	79
Sweden	84	71846±122548	96	59	43	60	74
Switzerland	12	96351±111170	12	13	67	83	92

Note: Table only includes countries with more than 5 city actors.

Supplementary Table 4. Grid Search Hyperparameters for the emissions prediction model

**Parameter**	**Search Range**	**Best Parameter Value**
max_depth	7,8,11,13,15	13
min_child_weight	1,3,5	1
eta	0.1,0.3,0.5,0.7,0.9	0.5
gamma	0.5,1,5	1
nrounds	999	40
early_stopping_rounds	5	-
objective	"reg:squarederror"	"reg:squarederror"

Supplementary Table 5. Grid Search Hyperparameters for comparison models

**Model**	**Parameter**	**Search Range**	**Best Parameter Value**
Random Forest	mtry	3,5,7	5
	min.node.size	1,3,5	1
	num.trees	700	700
	splitrule	"variance"	"variance"
SVM*	Cost	0.5, 1,5,7,10,15	15
	sigma	0.05,0.1,0.5	0.05

**Support Vector Machines with with Radial Basis Function Kernel*

Supplementary Table 6. Training and Test results of multiple comparison models**.**

**Model**	**Train_RMSE**	**Test_RMSE**	**Train_Rsquared**	**Test_Rsquared**
XGBoost-w/ NA	24202.05	155865.63	0.9995	0.8999

Random Forest	197987.79	157619.88	0.9799	0.9005
XGBoost-w/o NA	24632.40	171360.38	0.9995	0.8799
SVM	524193.32	174429.23	0.8527	0.8923
Multilinear Regression	476764.12	200363.18	0.8302	0.8479

Supplementary Table 7. Training and Test results of alternate model specifications.

**Variables**	**Best parameters [max_depth, min_child_Weight, eta, gamma, nrounds]**	**Train_RMSE**	**Test_RSME**	**Train_Rsquared**	**Test_Rsquared**
Hdd, odiac_c, gdp_pc, pm25, interpolated_population, interpolated_pop_density, DUSMASS, lat, lng	[13, 1, 0.5, 0.5, 38]	15620.88	432796.5	0.99	0.77
Hdd, odiac_c, gdp_pc, pm25, interpolated_population, interpolated_pop_density, DUSMASS, DMSMASS, BCSMASS, SO2SMASS, SO4SMASS, lat, lng	[9,1,0.3,1,52]	20630.16	448589	0.99	0.81
Hdd, odiac_c, gdp_pc, pm25, interpolated_population, lat, lng	[9, 1, 0.3, 1, 23]	38561.11	466055.1	0.99	0.82

References

OpenStreetMap contributors. (2015) Planet dump [Data file from February 15, 2021]. Retrieved from https://planet.openstreetmap.org.
