# Supplementary material for: Predicting European cities’ climate mitigation performance using machine learning
Source: Nat Commun. 2022 Dec 5;13:7487. doi: 10.1038/s41467-022-35108-5 (PMC9723121; doi:10.1038/s41467-022-35108-5)
Supplement: Supplementary file 3 — Reporting Summary [file 41467_2022_35108_MOESM3_ESM.pdf]

nature portfolio

Corresponding author(s): Angel Hsu

Last updated by author(s): Nov 7, 2022

Reporting Summary

Nature Portfolio wishes to improve the reproducibility of the work that we publish. This form provides structure for consistency and transparency in reporting. For further information on Nature Portfolio policies, see our Editorial Policies and the Editorial Policy Checklist.

Statistics

For all statistical analyses, confirm that the following items are present in the figure legend, table legend, main text, or Methods section.

n/a	Confirmed	
☐	☒	The exact sample size (*n*) for each experimental group/condition, given as a discrete number and unit of measurement
☐	☒	A statement on whether measurements were taken from distinct samples or whether the same sample was measured repeatedly
☐	☒	The statistical test(s) used AND whether they are one- or two-sided<br>*Only common tests should be described solely by name; describe more complex techniques in the Methods section.*
☐	☒	A description of all covariates tested
☐	☒	A description of any assumptions or corrections, such as tests of normality and adjustment for multiple comparisons
☐	☒	A full description of the statistical parameters including central tendency (e.g. means) or other basic estimates (e.g. regression coefficient) AND variation (e.g. standard deviation) or associated estimates of uncertainty (e.g. confidence intervals)
☐	☒	For null hypothesis testing, the test statistic (e.g. *F*, *t*, *r*) with confidence intervals, effect sizes, degrees of freedom and *P* value noted<br>*Give P values as exact values whenever suitable.*
☒	☐	For Bayesian analysis, information on the choice of priors and Markov chain Monte Carlo settings
☒	☐	For hierarchical and complex designs, identification of the appropriate level for tests and full reporting of outcomes
☒	☐	Estimates of effect sizes (e.g. Cohen's *d*, Pearson's *r*), indicating how they were calculated

Our web collection on statistics for biologists contains articles on many of the points above.

Software and code

Policy information about availability of computer code

Data collection	Data scraping and geospatial data processing were conducted using python (version 3.68), Beautiful Soup package (version 4.8.2), Beautiful Soup package (version 4.8.2), geopandas (version 0.9.0), rasterio version (1.0.21), and rasterstats (version 0.15.0), and the R statistical programming environment (version 3.6.2).
Data analysis	Statistical data analysis was conducted in the R statistical programming environment (version 3.6.2).The machine learning model was developed and conducted in R using the XGBoost package (version 1.6.0.1). Figures were made using ggplot2 data visualization package (version 3.3.6) and maps were made in QGIS (version 3.16). Custom functions written for the analysis are available on https://github.com/datadrivenenvirolab/citiesML.

For manuscripts utilizing custom algorithms or software that are central to the research but not yet described in published literature, software must be made available to editors and reviewers. We strongly encourage code deposition in a community repository (e.g. GitHub). See the Nature Portfolio guidelines for submitting code & software for further information.

Data

Policy information about availability of data

All manuscripts must include a data availability statement. This statement should provide the following information, where applicable:

- Accession codes, unique identifiers, or web links for publicly available datasets
- A description of any restrictions on data availability
- For clinical datasets or third party data, please ensure that the statement adheres to our policy

The data generated in this study have been deposited in the Data-Driven EnviroLab Dataverse repository (https://doi.org/10.15139/S3/NRJ5ZO). Raw data collected, processed and utilized for this study include: the Open-Data Inventory for Anthropogenic Carbon Dioxide (ODIAC) database (https://

doi.org/10.17595/20170411.001); NASA MERRA-2 monthly temperature product (https://doi.org/10.5067/KVIMOMCUO83U); NASA MERRA-2 monthly mean column mass density of aerosol components (black carbon, dust, sea salt, sulfate, and organic carbon), surface mass concentration of aerosol components (https://doi.org/10.5067/FH9A0MLJPC7N); Surface PM2.5 from the Atmospheric Composition and Analysis Group at Washington University at St. Louis (https://doi:10.1021/acs.est.1c05309); Gridded Population of the World dataset53 (http://dx.doi.org/10.7927/H4F47M2C) Globally-gridded gross domestic product (GDP) data from Kummu et al.54 (https://doi.org/10.1038/sdata.2018.4); Eurostat's Gross domestic product (GDP) at current market prices by NUTS 2 regions (http://data.europa.eu/88u/dataset/egT31kJF7IArVLXu1rTkQ); Kona et al.33 Global Covenant of Mayors, a dataset of greenhouse gas emissions for 6200 cities in Europe and the Southern Mediterranean countries (https://doi.org/10.5194/essd-13-3551-2021); other data for the EU Covenant of Mayors cities were collected from (https://www.covenantofmayors.eu/); Local Administrative Units from the Eurostat database55 (https://ec.europa.eu/eurostat/web/nuts/local-administrative-units); administrative boundaries of cities from OpenStreetMap (https://planet.openstreetmap.org); city centroids were extracted through Wikipedia's GeoHack website (https://www.mediawiki.org/wiki/GeoHack).

Field-specific reporting

Please select the one below that is the best fit for your research. If you are not sure, read the appropriate sections before making your selection.

☐ Life sciences ☒ Behavioural & social sciences ☐ Ecological, evolutionary & environmental sciences

For a reference copy of the document with all sections, see nature.com/documents/nr-reporting-summary-flat.pdf

Behavioural & social sciences study design

All studies must disclose on these points even when the disclosure is negative.

Study description	We develop a scalable, replicable machine learning methodology for evaluating the climate mitigation performance for more than 90,000 local and municipal actors in the European Union from 2001-2018. The data developed in this study are quantitative.
Research sample	All local administration units (LAUs) in the European Union as defined by the European Statistical Agency, including nearly 8,000 subnational government actors pledging voluntary climate action in the EU Covenant of Mayors for Climate and Energy (EUCoM). The data are representative since we did not sample but instead evaluated all local administrative units in Europe.
Sampling strategy	We included all subnational government actors pledging voluntary climate action in the EU Covenant of Mayors for Climate and Energy and included all LAUs in the European Union, therefore no sampling strategy was used since we considered all local administrative units in Europe and all participants in the EUCoM at the time of data collection.
Data collection	We collected and processed geospatial data from publicly-available sources, as noted in our Methods section, using python (version 3.68). The research team was aware of which cities were participants in the EUCoM, since we collected these cities' data from relevant databases (Kona et al., 2021) and from the website itself.
Timing	We collected data from the EUCoM website in February 2021, and geospatial data for the covariate predictors (GDP, fossil-fuel CO2 emissions, population, PM2.5) for all years that the data were available (year 2001 to 2018) in February 2021 and satellite remote sensing data for additional air pollution variables (dust, SO2, NO2, black carbon) were collected in May 2022. Satellite-derived temperature data was obtained in January 2022.
Data exclusions	We excluded any cities or local administrative units whose underlying spatial predictors did not meet data quality standards or which had missing data for emissions predictions.
Non-participation	No participants were involved in the study.
Randomization	Not relevant for our study since we were not conducting an experiment.

Reporting for specific materials, systems and methods

We require information from authors about some types of materials, experimental systems and methods used in many studies. Here, indicate whether each material, system or method listed is relevant to your study. If you are not sure if a list item applies to your research, read the appropriate section before selecting a response.

Materials & experimental systems

n/a	Involved in the study	
☒	☐	Antibodies
☒	☐	Eukaryotic cell lines
☒	☐	Palaeontology and archaeology
☒	☐	Animals and other organisms
☒	☐	Human research participants
☒	☐	Clinical data
☒	☐	Dual use research of concern

Methods

n/a	Involved in the study	
☒	☐	ChIP-seq
☒	☐	Flow cytometry
☒	☐	MRI-based neuroimaging
